# Risk of second cancer from scattered radiation of intensity-modulated radiotherapies with lung cancer

**DOI:** 10.1186/1748-717X-8-47

**Published:** 2013-03-04

**Authors:** Dong Wook Kim, Weon Kuu Chung, Dongoh Shin, Seongeon Hong, Sung Ho Park, Sung-Yong Park, Kwangzoo Chung, Young Kyung Lim, Dongho Shin, Se Byeong Lee, Hyun-ho Lee, Myonggeun Yoon

**Affiliations:** 1 Department of Radiation Oncology, Kyung Hee University Hospital at Gangdong, Seoul, Korea; 2 Department of Radiation Oncology, Kyung Hee University Medical Center, Seoul, Korea; 3 Department of medical Physics, Asan Medical Center, College of Medicine, University of Ulsan, Seoul, Korea; 4 Proton Therapy Center, McLaren Cancer Institute, Flint, USA; 5 Proton Therapy Center, National Cancer Center, Ilsan, Korea; 6 Department of Radiological Science, College of Health Science, Korea University, Jeongneung 3-dong, Seongbuk-gu, Seoul, Korea

**Keywords:** IMRT, VMAT, TOMOTHERAPY, Radio-photoluminescence, Secondary dose, OED

## Abstract

**Purpose:**

To compare the risk of secondary cancer from scattered and leakage doses following intensity-modulated radiotherapy (IMRT), volumetric arc therapy (VMAT) and tomotherapy (TOMO) in patients with lung cancer.

**Methods:**

IMRT, VMAT and TOMO were planned for five lung cancer patients. Organ equivalent doses (OEDs) are estimated from the measured corresponding secondary doses during irradiation at various points 20 to 80 cm from the iso-center by using radio-photoluminescence glass dosimeter (RPLGD).

**Results:**

The secondary dose per Gy from IMRT, VMAT and TOMO for lung cancer, measured 20 to 80 cm from the iso-center, are 0.02~2.03, 0.03~1.35 and 0.04~0.46 cGy, respectively. The mean values of relative OED of secondary dose of VMAT and TOMO, which is normalized by IMRT, ranged between 88.63% and 41.59% revealing 88.63% and 41.59% for thyroid, 82.33% and 41.85% for pancreas, 77.97% and 49.41% for bowel, 73.42% and 72.55% for rectum, 74.16% and 81.51% for prostate. The secondary dose and OED from TOMO became similar to those from IMRT and VMAT as the distance from the field edge increased.

**Conclusions:**

OED based estimation suggests that the secondary cancer risk from TOMO is less than or comparable to the risks from conventional IMRT and VMAT.

## Introduction

For earlier stages of lung cancer, the surgical resection has played the main role in its treatment. However there are some opportunities for radiation therapy when the tumor is located in the superior sulcus or is close to the critical normal organ, such as the esophagus and spinal cord, or for patients with positive lymph nodes. Furthermore, when the patient is in an in-operable situation according to their lung function, cardiac function, bleeding tendency or other reasons including the patient’s refusal for surgery, radiation therapy will be a beneficial option.

The radiation therapy technique has developed significantly over the last few decades. We have moved from simple 2 dimensional treatment to 3 dimensional conventional radiotherapy using the treatment fields to an increasingly conformal radiotherapy technique based on 3 dimensional computed tomography (CT) information such as three dimensional conformal therapy (3DCRT) and intensity modulated radiotherapy (IMRT) [1-7]. Recently, volumetric modulated arc therapy (VMAT) and helical tomotherapy (TOMO) have been introduced which can deliver rotational intensity-modulated therapy with more degrees of freedom of gantry speed, multileaf collimator (MLC) leaf motion and dose rates to maximize the target conformity and sparing the normal tissue dose [8-13]. In this study, we estimate the secondary cancer risk of normal organs which are out-of-field for IMRT, VMAT and TOMO with lung cancer patients.

In general, when tumors in cancer patients during the radiation treatment are exposed to high doses which are prescribed for a definitive or palliative goal, the surrounding normal tissues are exposed to intermediate doses which is due to the primary radiation in the beam path. Therefore, the main goal of the treatment planning is finding the right option to satisfy two conflicting priorities such as reducing the exposed dose into the surrounding normal organ and focusing the prescription dose into a target volume. However, out-of-field exposure is another interesting item to be concern. The rest of the body is also exposed to low doses during the radiation treatment which is due primarily to out-of-field radiation resulting from scattering and leakage. Therefore, it will be interesting to measure and estimate the exposed dose for normal organs in out-of-field regions. Furthermore, the evaluation of secondary cancer risk from the out-of-field dose would be interesting, too.

To date, there have been many measurements and calculations of secondary scattered dose and secondary cancer risk [14-22]. In 2003, Hall E. and Wuu C. S. reported the radiation induced second cancers. They are concerned that the secondary cancer risk may be increased by moving from 3DCRT to IMRT which use more fields and monitor units to increase the exposed normal tissue volume by low dose and total body exposure due to leakage radiation. They reported that IMRT induces almost double the incidence of second malignancies compared with 3DCRT [14]. Kim S. et al. presented the secondary radiation doses of intensity-modulated radiotherapy and proton therapy in patients with lung and liver cancer [15]. They measured the secondary scattered dose of IMRT at 20–50 cm from iso-center, ranging from 5.8 and 1.0 mGy per Gy. However, they did not present the calculation results of secondary risk from their measurement [16].

In this study, we compared the secondary cancer risk by out-of-field radiation for three treatment modalities using the concept of organ equivalent dose (OED) for radiation-induced cancer.

## Methods and materials

### Patient data and treatment planning

We randomly selected five patients with lung cancer who were to be treated with IMRT at Kyunghee University hospital at Gangdong. All of these patients had undergone treatment planning CT scans (Brilliance CT Big Bore Oncology; Philips Medical System, The Netherlands) of the chest for identification of targets and normal neighboring organs. An Eclipse (Varian Medical Systems, Palo Alto, CA, USA) and Hi-Art (TomoTherapy, Madison, WI, USA) planning system were used to plan IMRT, VMAT and TOMO for these patients. As shown as Table 1, the patient group is consisted of three male and two female patients. The age range was from 56 to 71 years old with an average age of 67. All patients are stage III of non small cell lung cancer (NSCLC) cases and PTV volumes are varied from 64 to 890 cc.

**Table 1:** Patient information

**ID**	**Sex**	**Age**	**Disease**	**Stage**	**PTV volume (cm**^**3**^**)**
1	Male	69	NSCLC	III	384
2	Female	71	NSCLC	III	341
3	Female	56	NSCLC	III	64
4	Male	70	NSCLC	III	210
5	Male	69	NSCLC	III	890

**Figure 1:**
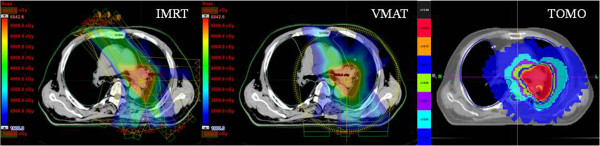
**Treatment plan for patient #4 with different modalities; IMRT, VMAT and TOMO.** The prescription dose 62.5 Gy with 25 fractions.

The targets in the 5 lung cancer patients were defined in accordance with the report of the International Commission on Radiation Units and Measurements (ICRU 50). 4 dimensional computed tomography image was obtained during the CT scan by using a Philips brilliant big bore CT with Varian real-time patient monitoring system (RPMS). Particularly, a gross tumor volume (GTV) encompassed all detectable tumors and lymph nodes that were at least 1 cm in short-axis diameter, as observed on CT scans. The clinical target volume (CTV) included the GTV and uninvolved mediastinal and ipsilateral hilar nodes. Planning target volume (PTV) included the CTV plus a 7–10 mm margin. Each patient received a total dose of 50–63 Gy, using different fractionation schemes, to the iso-center. The prescribed dose was specified at the ICRU reference point (iso-center) of the PTV. All treatment plans used four to nine beams for IMRT, single or double arcs for VMAT and a helical beam for TOMO. As an example, Figure 1 shows the treatment plans of patient 4 with different modalities; IMRT, VMAT and TOMO.

### Calibration of the radio-photoluminescence glass dosimeter

A radio-photoluminescence glass dosimeter (RPLGD) is newly introduced, as a substitution of the themoluminescence dosimeter (TLD) or other, which was commonly used for in-vivo measurement [23-27]. In this study, we used commercially available RPLGD (GD-302M, Asahi Techno Glass Co., JAPAN). An RPLGD measured the absorbed dose by counting the orange light (500 ~ 700 nm) from the dosimeter, when 365 nm of mono-energetic light was exposed on the irradiated dosimeter. RPLGD has relatively good reproducibility at about 1% and low energy dependency, at higher than 200 keV energy [23-27]. In addition, RPLGD has relatively small incident beam angular dependency, and low toxicity inside the human body, compared with a TLD or optically stimulated luminescence dosimeter (OSLD) [28-30]. A geometrical shape of RPLGD is a rod with 0.15 cm of the diameter and 0.85 cm length.

For estimating the dose response of RPLGD, 10 × 10 cm [2] open field photon beam was exposed into RPLGD at a 10 cm depth, and 100 cm of Source Surface Distance (SSD). The reproducibility of RPLGD is estimated by calculating the standard deviation of the dose measurements, which the photon beam exposed 3 times into the same detector. Also, the deviations of each RPLGD detector are measured to characterize each RPLGD.

### Measurement of secondary dose during IMRT, VMAT and Tomotherapy treatment

**Figure 2:**
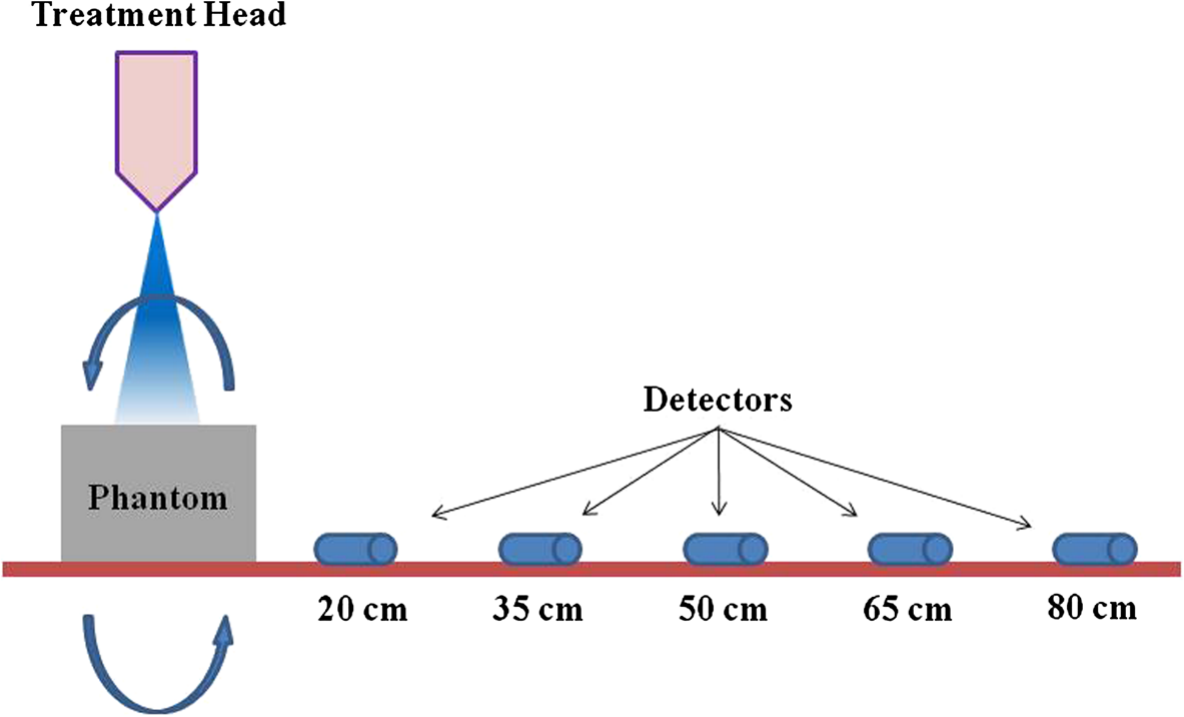
**Secondary scattered dose measurement set up.** Measuring positions are 20, 35, 50, 65 and 80 cm from the iso-center. Two RPLGDs are positioned at each measuring point.

In all treatments, a photon of 6 MV energy (Clinac 21iX; Varian Medical Systems, USA) was used for IMRT and VMAT. Secondary radiation was assessed by measuring the ionization of the photon beam as a function of distance from the iso-center, because the contribution of the secondary neutron dose is negligible in 6MV photon beams. These measurements were performed using two RPLGDs set at distances 20, 35, 50, 65 and 80 cm from the beam iso-center on a solid phantom at SAD 100 cm as shown as Figure 2.

Secondary photon doses were measured using an RPLGD on the surface of couch table; thus, the secondary dose, measured at various distances from the iso-center, was the maximum possible dose at that distance and decreased with depth in the body. Therefore, the actual doses at certain body depths at each distance from the iso-center will be smaller than the measured doses.

### Cancer risk estimation attributable to secondary doses

From statistical data on atomic bomb survivors and medically exposed patients, Schneider et al. proposed use of the “Excess Absolute Risk” (EAR) index per 10,000 persons per year to estimate the radiation-induced carcinogenesis.

(1) $$\begin{array}{l} \text{EA}R\;\left(D,\;s,\;e,\;a\right)\;=\;\beta \cdot f\left(D\right)\cdot g\left(s,\;e,\;a\right)\\ \;=\;\beta \cdot f(D)\cdot [\text{exp}(\gamma \_e(e-37)\\ \;+\;\gamma \_a\;\text{ln}(a/46))(1\pm s) \end{array}$$

where *β* is the initial slope, *f(D) *is a function of dose, and *g(s, e, a)* is a modifying function of population dependent variables such as gender (*s*), age at exposure (*e*), and age attained (*a*), respectively. In Eq. (1), the fit parameters are gender (*s*) averaged (+ for female, - for male) and mean age at exposure (*e*) of 37 years old and attained age of 46 years old. The function of dose *f(D)*, which is the dose-dependent portion of Eq. (1), is OED, when averaged over the whole body of volume. It should be noted that OED, a dose–response-weighted dose variable, is proportional to cancer risk in a defined population (same gender, same age of exposure, and same attained age).

The OED calculation based on the linear dose–response model:

(2) $$\;\text{OED}\;=\;\frac{1}{V}\sum_{i}V_{i}D_{i}$$

or a linear-exponential (bell-shape) dose–response model:

(3) $$\;\text{OED}\;=\;\frac{1}{V}\;\sum_{i}V_{i}D_{i}\;\text{exp}\left(-{\mathrm{\alpha D}}_{i}\right)$$

or a plateau dose–response model:

(4) $$\;\text{OED}\;=\;\frac{1}{V}\sum_{i}V_{i}\left(\frac{1-\text{exp}\left(-\sigma D_{i}\right)}{\sigma }\right)$$

where *V* is the whole body volume, *V*_*i*_ is a volume and *D*_*i*_ is the dose elements, respectively. In these models, parameters such as *α* and *σ* are used to determine the dose–response curve for specific organs. We compared the radiation-induced secondary cancer risk resulting from IMRT, VMAT and TOMO for NSCLC patients, based on analysis of OEDs.

## Results and discussion

Table 2 shows a comparison of treatment plans for different modalities. For IMRT, 4 to 9 fields are used and the monitor units per Gy ranged from 245 to 1049 MU/Gy. For VMAT, 1 or 2 full arcs are used and the monitor unit per Gy ranged from 245 to 430 MU/Gy. For TOMO, the monitor units per Gy ranged from 1025 to 2021MU/Gy. The monitor unit per Gy is increased by increasing number of fields (or Arcs) and PTV size. Therefore, patient 3 has a relatively lower monitor unit per Gy than other cases as shown in Table 2. In addition, the monitor unit per Gy is dependent on the modality. VMAT has relatively small monitor units as 0.78±0.35 times of IMRT. TOMO has most of the higher monitor units compared to others at 3.69±0.92 times the large monitor units of IMRT. As confirmed in former reports, VMAT presents most of the small monitor units comparing to IMRT and TOMO thus, it facilitates shorter treatment time and monitor units which are related to patient immobilization and machine maintenance.

**Table 2:** Treatment planning information

**ID**	**Modality**	**# of fields (or arcs)**	**MU/Gy**
1	IMRT	4	487
	Arc	2	397
	Tomo	n/a	2021
2	IMRT	5	437
	Arc	1	245
	Tomo	n/a	1831
3	IMRT	5	245
	Arc	2	353
	Tomo	n/a	1025
4	IMRT	7	469
	Arc	1	323
	Tomo	n/a	1921
5	IMRT	9	1049
	Arc	2	430
	Tomo	n/a	1940

**Figure 3:**
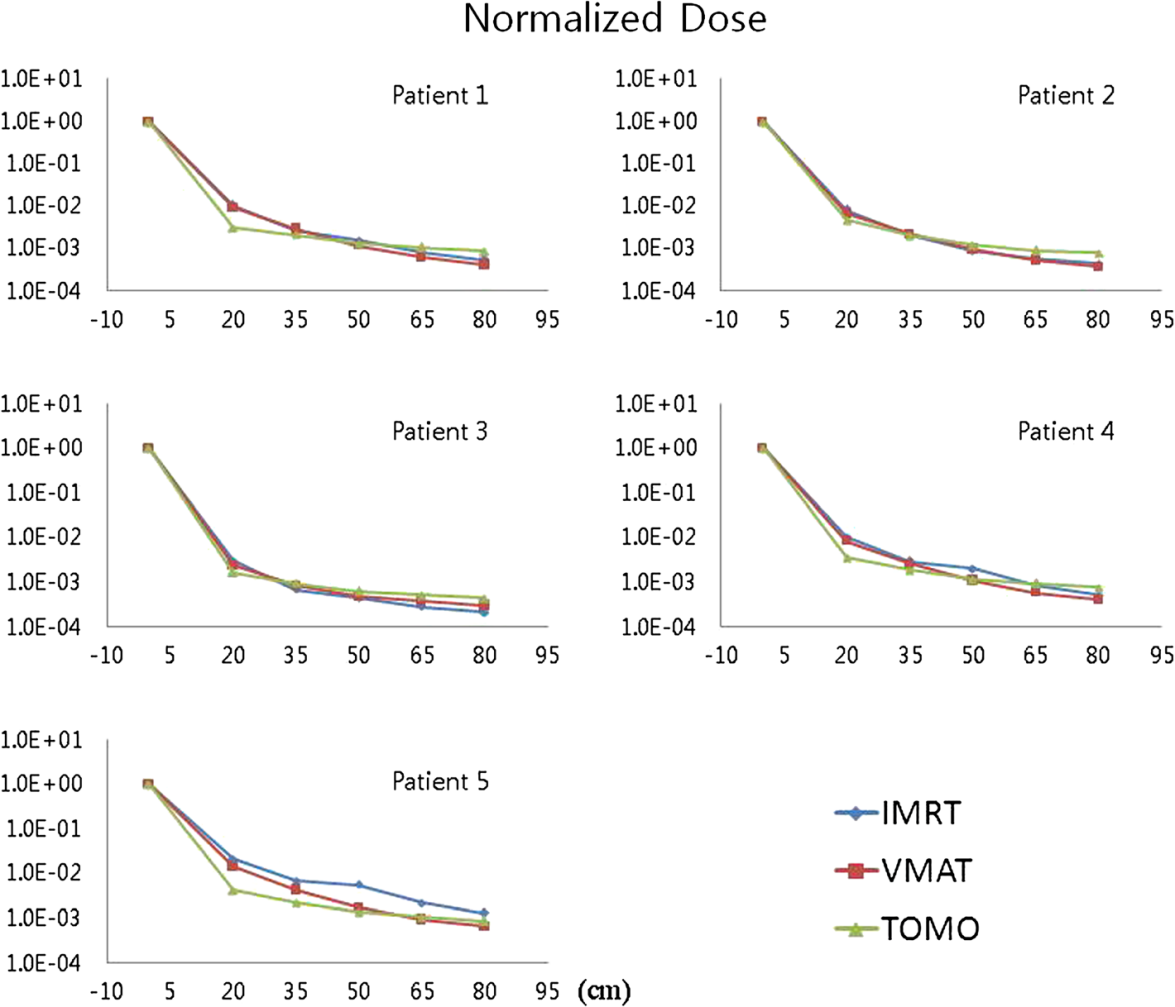
**The secondary scattered dose measurements of five patients for IMRT (blue, diamond), VMAT (red, square) and TOMO (brown, triangle) at each distance from the iso-center.** All data are normalized by prescription dose.

**Figure 4:**
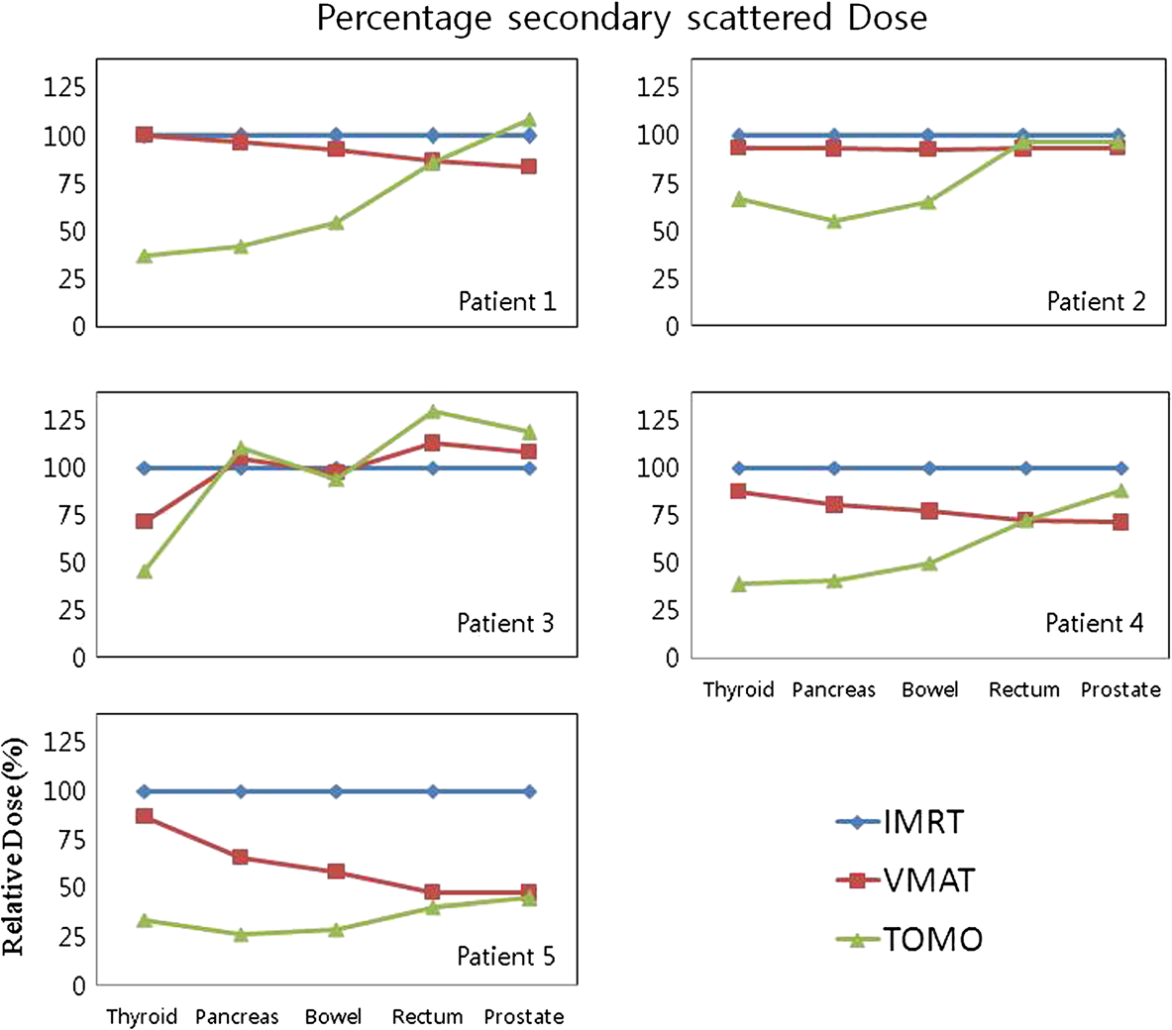
**The percentage secondary scattered dose measurements of five patients for IMRT (blue, diamond), VMAT (red, square) and TOMO (brown, triangle) at each distance from the iso-center.** VMAT and TOMO result is normalized by IMRT result.

Table 3 and Figure 3 show the secondary scattered dose measurement of five patients for IMRT, VMAT and TOMO. In Table 3, the secondary scattered dose measurement as percentage of prescription doses at each point are shown for each modality and patient. The average percentage scattered dose for prescription dose for five patients at 20, 35, 50, 65 and 80 cm from the iso-center is 1.04±0.56, 0.30±0.20, 0.21±0.18, 0.09±0.06 and 0.06±0.04% for IMRT, 0.80±0.36, 0.25±0.11, 0.11±0.04, 0.06±0.02 and 0.04±0.01% for VMAT, 0.34±0.10, 0.18±0.04, 0.11±0.03, 0.09±0.02 and 0.07±0.02% for TOMO. The secondary scattered dose is decreased as the distance from the in-field region is increased. In addition, the secondary scattered dose depends on the modality. TOMO (VMAT) has about 30% (80%) of secondary scattered dose comparing to secondary scattered dose of IMRT at 20 cm distance from the iso-center. And the secondary scattered dose decreases as the distance from the iso-center increases resulting from the fact that the secondary scattered dose at 80 cm distance from the iso-center is about 20 times lower than the dose measurement at 20 cm for IMRT and VMAT. Also we found that the decreasing slope of the secondary scattered dose of IMRT and VMAT is similar and more steep than TOMO. Therefore, TOMO has a relatively low secondary dose around the target area in spite of the fact that the monitor unit of TOMO treatment cases are higher and close to the values of IMRT and VMAT, as distance is increased from the iso-center as shown as in Figure 4. TOMO has a 22 cm of tungsten shielding in primary jaws, the MLC, and head shielding [31]. Because of the maximize beam shielding for radiation leakage in TOMO comparing to the conventional linear accelerators to give rise to lower scattered dose especially around the target area.

**Table 3:** At each points, the secondary scattered dose measurements as percentage of prescription dose

**ID**	**Modality \ Distance**	**20 cm**	**35 cm**	**50 cm**	**65 cm**	**80 cm**
1	IMRT	1.0282	0.2556	0.1517	0.0779	0.0522
		±0.0064	±0.0018	±0.0049	±0.0008	±0.0003
	Rapidarc	0.9218	0.2883	0.1164	0.0617	0.0406
		±0.0089	±0.0048	±0.0014	±0.0023	±0.0003
	Tomotherapy	0.2989	0.2087	0.1305	0.1054	0.0863
		±0.0038	±0.0007	±0.0005	±0.0015	±0.0007
2	IMRT	0.8028	0.2107	0.0888	0.0572	0.0436
		±0.0237	±0.0060	±0.0013	±0.0026	±0.0001
	Rapidarc	0.6727	0.2132	0.0949	0.0530	0.0364
		±0.0074	±0.0023	±0.0012	±0.0007	±0.0004
	Tomotherapy	0.4626	0.2011	0.1196	0.0883	0.0780
		±0.0036	±0.0048	±0.0016	±0.0081	±0.0002
3	IMRT	0.3038	0.0682	0.0449	0.0283	0.0205
		±0.0040	±0.0009	±0.0002	±0.0008	±0.0003
	Rapidarc	0.2312	0.0827	0.0480	0.0374	0.0297
		±0.0032	±0.0015	±0.0008	±0.0003	±0.0001
	Tomotherapy	0.1634	0.0946	0.0623	0.0507	0.0438
		±0.0022	±0.0014	±0.0004	±0.0005	±0.0011
4	IMRT	1.0168	0.2854	0.2034	0.0845	0.0516
		±0.0355	±0.0067	±0.0013	±0.0008	±0.0007
	Rapidarc	0.8301	0.2491	0.1071	0.0582	0.0402
		±0.0037	±0.0039	±0.0046	±0.0003	±0.0008
	Tomotherapy	0.3453	0.1828	0.1128	0.1920	0.0769
		±0.0063	±0.0021	±0.0011	±0.0023	±0.0010
5	IMRT	2.0248	0.6576	0.5496	0.2158	0.0280
		±0.0323	±0.0456	±0.0040	±0.0019	±0.0020
	Rapidarc	1.3508	0.4202	0.1727	0.0932	0.0635
		±0.0163	±0.0074	±0.0032	±0.0044	±0.0005
	Tomotherapy	0.4185	0.2179	0.1312	0.1032	0.0850
		±0.0058	±0.0097	±0.0007	±0.0014	±0.0008

**Figure 5:**
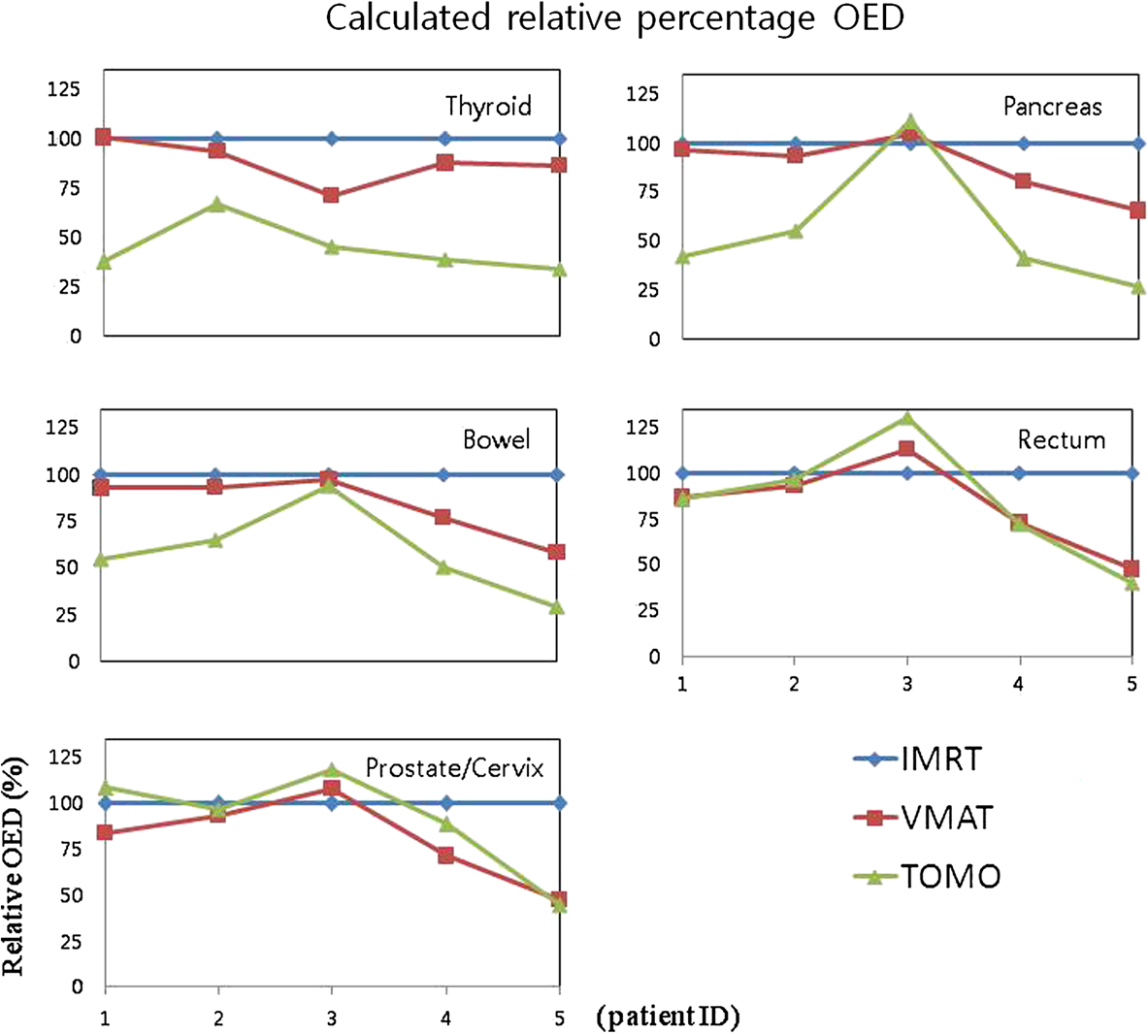
**The calculated relative percentage of OED of five interesting organs (Thyroid, Pancreas, Bowel, Rectum and Prostate/Cervix) for IMRT (blue, diamond), VMAT (red, square) and TOMO (brown, triangle) at each distance from the iso-center.** VMAT and TOMO result is normalized by IMRT result.

For VMAT and TOMO, the calculated relative percentage OED which is normalized by OED of IMRT for each organ, is presented in Table 4 and Figure 5. As predicted from the measurements of the secondary scattered dose, TOMO has a relatively low OED for most of the organs compared to the other modalities and this OED difference from modality decreases when the position of the organ gets further away from the field edge. Only in the case of patient 3 who has a relatively low PTV volume (69 cm [3]), the modality dependence for the OED did not show. Because of the statistical limitation, it is not clear if there is any correlation between the secondary scattered dose (or OED) and the PTV volume. Therefore it is necessary to measure and estimate the secondary scattered dose (or OED) for different PTV volumes with more statistics in the future, to elucidate the correlation of the source of secondary scattered dose difference deriving from the distance from the iso-center (or field edge), treatment modality, PTV volume, and other factors.

**Table 4:** Relative percentage OED which is normalized by OED of IMRT

**ID**	**Modality \ Site**	**Thyroid**	**Pancreas**	**Bowel**	**Rectum**	**Prostate/Cervix**
1	IMRT	100.00±0.64	100.00±0.64	100.00±0.18	100.00±0.49	100.00±0.49
	Rapidarc	100.47±1.10	96.63±1.06	92.66±0.48	86.66±0.44	84.06±0.43
	Tomotherapy	37.53±0.28	42.19±0.31	54.67±0.10	85.77±0.42	108.61±0.53
2	IMRT	100.00±2.37	100.00±2.37	100.00±0.60	100.00±0.13	100.00±0.13
	Rapidarc	93.86±2.33	93.26±2.31	92.91±0.59	93.14±0.16	93.45±0.16
	Tomotherapy	66.59±1.59	55.35±1.32	65.36±0.50	96.76±0.20	96.91±0.20
3	IMRT	100.00±0.40	100.00±0.09	100.00±0.09	100.00±0.02	100.00±0.02
	Rapidarc	71.26±0.37	105.03±0.18	97.48±0.17	113.00±0.09	108.42±0.09
	Tomotherapy	45.33±0.21	110.78±0.18	93.82±0.15	130.36±0.06	118.89±0.05
4	IMRT	100.00±3.55	100.00±3.55	100.00±0.67	100.00±0.13	100.00±0.13
	Rapidarc	87.51±3.12	80.51±2.88	77.15±0.60	72.83±0.35	71.40±0.34
	Tomotherapy	38.94±1.40	41.04±1.48	50.34±0.35	72.60±0.12	88.55±0.15
5	IMRT	100.00±0.08	100.00±3.23	100.00±4.56	100.00±0.40	100.00±0.19
	Rapidarc	86.57±1.42	65.38±2.37	58.22±2.69	47.52±0.24	47.39±0.23
	Tomotherapy	33.71±0.20	26.57±0.87	29.12±1.36	40.31±0.16	44.77±0.11

## Conclusions

We compared secondary scattered doses and OED which is related with radiation induced secondary malignancy risk. We found that the secondary dose depended on the distance from the iso-center and their modalities. The secondary dose and OED from TOMO is less than or compatible to the secondary dose from conventional IMRT and VMAT. The secondary dose and OED from TOMO became similar to IMRT and VMAT as the distance from the field edge increased.

## Consent

Written informed consent was obtained from the patient for publication of this report and any accompanying images.

## Competing interests

The authors declare that they have no competing interest.

## Authors’ contribution

DWK and MY did design, process and write this article. WKC and HH were provided the patient data and clinical support. DS and KC participated in tomotherapy planning and measurement. SYP and SP participated in IMRT planning and measurement. YKL and DS participated in the VMAT planning and measurement. SBL and HL participated the RPLGD reading and the EUD calculation. All authors read and approved the final manuscript.
